# The Effects of a Gentle Yoga Program on Sleep, Mood, and Blood Pressure in Older Women with Restless Legs Syndrome (RLS): A Preliminary Randomized Controlled Trial

**DOI:** 10.1155/2012/294058

**Published:** 2012-02-28

**Authors:** Kim E. Innes, Terry Kit Selfe

**Affiliations:** ^1^Department of Community Medicine, West Virginia University School of Medicine, P.O. Box 9190, Morgantown, WV 26506-9190, USA; ^2^Center for the Study of Complementary and Alternative Therapies, University of Virginia Health System, P.O. Box 800782, McLeod Hall, Charlottesville, VA 22908-0782, USA

## Abstract

*Objective*. To examine the effects of yoga versus an educational film program on sleep, mood, perceived stress, and sympathetic activation in older women with RLS. *Methods*. Participants were drawn from a larger trial regarding the effects of yoga on cardiovascular disease risk profiles in overweight, sedentary postmenopausal women. Seventy-five women were randomized to receive either an 8-week yoga (*n* = 38) or educational film (*n* = 37) program. All 75 participants completed an RLS screening questionnaire. The 20 women who met all four diagnostic criteria for RLS (*n* = 10 yoga, 10 film group) comprised the population for this nested study. Main outcomes assessed pre- and post-treatment included: sleep (Pittsburgh Sleep Quality Index), stress (Perceived Stress Scale), mood (Profile of Mood States, State-Trait Anxiety Inventory), blood pressure, and heart rate. *Results*. The yoga group demonstrated significantly greater improvements than controls in multiple domains of sleep quality and mood, and significantly greater reductions in insomnia prevalence, anxiety, perceived stress, and blood pressure (all *P*'s≤0.05). Adjusted intergroup effect sizes for psychosocial variables were large, ranging from 1.9 for state anxiety to 2.6 for sleep quality. *Conclusions*. These preliminary findings suggest yoga may offer an effective intervention for improving sleep, mood, perceived stress, and blood pressure in older women with RLS.

## 1. Introduction

Restless legs syndrome (RLS) is a distressing and potentially debilitating sleep disorder, affecting up to 29% of U.S. and European general adult populations, and an estimated average of 19.5% of primary care patients [1]. RLS is characterized by a compelling urge to move the legs, usually accompanied by uncomfortable sensations in the legs, that: begins or worsens during periods of inactivity, is worse during the evening and nighttime hours, and is partially or totally relieved by movement [2]. Prevalence increases with age, and RLS is approximately twice as common in women as in men [1]. RLS has been repeatedly associated with significant reductions in quality of life comparable to or worse than those reported in Parkinson's disease, diabetes, stroke, and other serious chronic disorders [3–7]. RLS has also been linked to substantial impairment in sleep, mood, and health and is associated with significant societal and economic burden [4, 5, 7].

While RLS is considered a neurological disorder, the etiology of RLS remains poorly understood [8–10]. Defects in dopamine function and iron metabolism have been considered key factors in the pathogenesis of RLS for decades [11–14], based largely on clinical observations [14–18], although evidence remains inconclusive [8, 15, 19, 20]. An emerging body of evidence supports a potential role for autonomic and metabolic dysfunction. Recent studies have linked RLS to cardiovascular disease (CVD), as well as to key components of the metabolic syndrome, including diabetes, obesity, hypertension, and dyslipidemia, conditions associated with both autonomic and metabolic dysregulation [21]. RLS is characterized by elevated nocturnal blood pressure and heart rate [22–24], increased nocturnal hypothalamic-pituitary-adrenal (HPA) axis activation [25], elevated sympathetic activation, and reduced parasympathetic tone [26, 27], suggesting that autonomic and HPA axis dysfunction may in part underlie the development and/or progression of RLS and could help explain the observed association of RLS to CVD and related disorders [21].

There is no cure for RLS. Existing treatments are aimed at symptom reduction and include dopaminergic agents, opioids, sedative hypnotics, anticonvulsants, and benzodiazepines. Unfortunately, these medications can have serious side effects. For example, common side effects of dopaminergic agents, considered the first-line pharmacologic treatment for RLS sufferers [4, 16, 28], include rebound and augmentation of symptoms, dyskinesia, nausea and vomiting, hypotension, impulse control disorders, dizziness, and insomnia or drowsiness [7, 29–31]; common side effects of other RLS medications include potential for dependence, confusion, blurred vision, coordination problems, and other adverse sequellae [7, 29, 30]. These effects can be particularly problematic in older populations [32, 33], who also suffer disproportionately from RLS [29, 34]. In addition, the benefits of these medications may diminish over time [35, 36], leaving patients with few treatment options.

Given the substantial drawbacks of these pharmacologic treatments, investigation of safe, nonpharmacologic therapies that address apparent underlying risk factors is needed. Yet research remains very sparse. For example, there have to date been only three small trials examining the potential benefits of lifestyle or behavioral interventions for individuals suffering from RLS. These include two studies evaluating the effects of a 12–16 week exercise program versus usual care on RLS symptoms [37, 38], and a recent pre-post trial assessing the effects of cognitive behavioral therapy (CBT) in adults with primary RLS and mild-moderate psychosocial impairment [39]. Investigators reported significant improvements in RLS-related outcomes relative to the control group [37, 38] or baseline [39], suggesting that nonpharmacologic interventions may benefit those suffering from RLS.

Yoga, an ancient mind-body discipline that is increasingly popular in the U.S. [40], may represent a particularly promising nonpharmacologic therapy for RLS. Accumulating evidence from controlled trials suggests yoga can reduce blood pressure [41], improve glucose tolerance [41–43], lipid profiles [41, 44, 45], body composition [41, 45], and autonomic function [42, 44, 45], enhance mood [46–49], and improve sleep [48, 50, 51], factors linked to both RLS and CVD risk [8, 9, 16, 21]. In addition, studies suggest yoga can decrease muscular excitability and induce more rapid muscle relaxation [52], factors that have also been implicated in RLS [53, 54]. However, while yoga and other relaxation therapies are often recommended for RLS patients [55, 56], clinical trials are lacking. In this small, nested RCT, we investigated the effects of yoga on sleep, mood, stress, and associated outcomes in older women with RLS.

## 2. Methods

Participants for this study were drawn from a feasibility study regarding the effects of yoga on CVD risk profiles in sedentary, overweight postmenopausal women [57]. In this larger RCT, 75 women aged 45–79 years were recruited from the community using a combination of flyers, brochures, and newspaper advertisements. Eligible participants were nonsmoking women at least 45 years of age who were postmenopausal (≥12 months amenorrheic), physically inactive (exercising less than 20 minutes, 3 times per week), and overweight (body mass index (BMI, calculated as kg/m^2^) ≥25, and/or waist circumference ≥88 cm) or had a first-degree relative with diabetes or essential hypertension. Excluded were women who had practiced yoga within the last year, had uncontrolled hypertension, had been diagnosed with sleep apnea or with diabetes, cancer, heart failure, or other serious chronic disorders, or had any orthopedic, neurological, or other condition that might prevent them from safely completing an 8-week yoga program. Following enrollment, screening, and baseline assessment, eligible participants were randomized, using a computer-generated randomization list provided by a statistician not associated with the study, to receive either a gentle 8-week yoga program (*n* = 38) or an 8-week educational film program (*n* = 37). Each participant was administered her treatment assignment sequentially in order of enrollment, via coded opaque envelopes containing general study instructional materials, a welcome letter, and information pertinent to the yoga or educational film program. The study was approved by the University of Virginia Health Sciences Research Institutional Review Board, and all participants provided informed consent prior to study enrollment.

### 2.1. RLS and Outcome Assessment

 Participants completed an RLS screening questionnaire at baseline. The questionnaire was adapted by the authors from the Athens Sleep Center Screening Questionnaire for RLS (http://www.athenssleepcenter.com/PDF/AScreeningQuestionaireRLS.pdf) to incorporate the established four criteria for RLS established by the International Restless Legs Syndrome Study Group (IRLSSG) [2]. Participants were asked if they ever experienced an urge to move with uncomfortable/funny (e.g., tingling, creeping, crawling) sensations in the legs, and if these feelings began or worsened when lying or sitting, were worse in the evening/night, and were relieved at least in part by movement (e.g., wiggling feet, toes, or walking). The questionnaire also included an item regarding frequency of symptoms (never, occasionally (<1x/month), sometimes (1–3x/month), frequently (1-2x/week to daily), or only in the past). Those participants endorsing all 4 diagnostic criteria and reporting symptoms at least once per month (*N* = 20) were considered to meet diagnostic criteria for RLS and were included in the present study.

Participants underwent a comprehensive assessment at baseline and following the 8-week intervention period. All assessments were conducted by experienced General Clinical Research Center and university clinical laboratory staff blinded to participant treatment assignment. Detailed baseline information was gathered on medical history, demographic characteristics, and lifestyle factors. *Primary outcomes* for this substudy included well-validated self-report measures of sleep (Pittsburgh Sleep Quality Index (PSQI)) [58], stress (Perceived Stress Scale (PSS)) [59], mood (Profile of Mood States (POMS)) [60], and anxiety (State-Trait Anxiety Inventory (STAI)) [61], as well as indices of sympathetic activation (blood pressure, heart rate). Clinical insomnia was defined as a score of greater than 5 on the PSQI [58, 62]. Blood pressure and heart rate were measured in a supine position following a 5-minute rest period; measurements were taken 3 times and averaged for a final score.

Additional outcomes included indices of abdominal adiposity (waist circumference (cm) and BMI (kg/m^2^)). At baseline, all women were screened for prediabetes (fasting glucose 100–125 mg/dL)/diabetes (fasting glucose ≥126 mg/dL), and serum levels of ferritin were measured. Blood samples were collected in the morning following an overnight fast and drawn by a trained phlebotomist using Vacutainer tubes. Blood samples (ferritin) were stored at −70°C until assay. Glucose was assessed using a Beckman glucose analyzer. Ferritin levels were measured using the ARCHITECT ferritin assay, a Chemiluminescent Microparticle Immunoassay (CMIA) for the quantitative determination of ferritin in human serum and plasma.

To assess the possible influence of physical activity and social support on change in outcomes over time, factors implicated in RLS [63] and strongly linked to both sleep and mood [64], participants completed the Physical Activity Scale for the Elderly (PASE) [65] and the Duke Social Support Index (abbreviated form [66]) pre- and posttreatment. To measure expectations of benefit, a brief treatment expectancy questionnaire was also administered to all participants. In addition, all subjects were asked to complete a short, anonymous exit questionnaire regarding their experience with the study.

### 2.2. Intervention

 Each group (yoga and film intervention) attended a 90-minute class twice weekly for 8 weeks. Classes for the two programs were scheduled at the same times and in similar settings. Class size in both groups was limited to no more than 14 participants. Upon completing the study, all participants were given $150 for their time and travel expenses. Film group participants also received the yoga home practice materials, as well as coupons to attend local Iyengar yoga classes. Similarly, yoga group participants were also offered the opportunity to view the educational films following completion of the final assessment.

#### 2.2.1. Yoga Intervention

 Participants randomized to the yoga program completed a gentle Iyengar yoga program specifically designed for older, sedentary adults. In addition to attending classes, participants were asked to complete at least 30 minutes of home practice on nonclass days, with the aid of a DVD and a booklet illustrating the yoga home practice routines. Designed and taught by a senior Iyengar yoga instructor with over 30 years of experience, the yoga program included the following 23 active and restorative poses (asanas): Mountain (Tadasana and Urdhva hastasana in Tadasana); Standing wide apart legs (Prasarita padottanasana); Warrior I and II (Virabhadrasana I and II); Fierce or chair pose (Utkatasana); Extended hands and feet pose (Utthita hasta padasana); 1/2 forward bend (Ardha uttanasana); Triangle (Utthita trikonasana); Reverse triangle (Parivrtta trikonasana); Downward facing dog (Adho mukha svanasana); Marichi's pose (Marichyasana I and III, Utthita marichyasana); Seated twist in chair (Bharadvajasana); One leg straight forward spinal stretch (Janu sirsasana); Seated wide angle (Upavista konasana and Parsva upavista konasana); Seated bound angle (Baddha konasana); Extended legs up the wall (Urdhva prasarita padasana); Lying down holding big toe (Supta padangusthasana); Turned around belly (Jathara parivartanasana); Supported boat (Navasana); Supported bridge (Setu bandha sarvangasana); Crossed legs back arch (Supta swastikasana); Lying down bound angle (Supta baddha konasana); Reclined mountain (Supta tadasana); and Corpse pose (Savasana). Poses were modified and props (blankets, chairs, and straps) used as needed to allow participants to perform the sequences easily and safely. Each session began with a simple yogic centering and breathing exercise and ended with a 10–15 minute guided supine relaxation practice (Savasana).

#### 2.2.2. Educational Film (Control) Intervention

 This standardized educational film program, described in detail elsewhere [57], was designed to be easily replicable and to ensure comparability of staff attention, class time, and social interaction. Each class began with a brief meet and greet period, followed by viewing of an educational film chosen for its relevance and interest to our study population, then concluded with a 10–15 minute group discussion facilitated by a health professional with expertise in complementary and alternative therapies and women's health. To increase credibility and reduce participant bias, the educational film program was presented to potential participants as an informative, engaging, and relaxing alternative to the yoga classes.

### 2.3. Adherence and Adverse Events

 Attendance at both the yoga and the film classes was recorded by the respective instructors. Participants in the yoga class also completed a check sheet and log after each home practice session, indicating the number of minutes practiced and any comments they might have regarding the session. Homework logs were turned in to the instructor at the first group class each week.

Adverse events were tracked via weekly review of participant yoga logs. At the beginning of each class, the yoga instructor also queried participants regarding potential problems; these were likewise recorded. In addition, participants were encouraged to contact study investigators and/or staff regarding any potential concerns.

### 2.4. Statistical Analysis

 Data were analyzed using PASW v. 18. Differences between those who did versus did not meet criteria for RLS at baseline, and between yoga and control group participants at baseline were evaluated using chi-square (for categorical variables), student independent samples t tests (for continuous variables with a normal distribution), or Mann-Whitney *U* tests (for ordinal variables or continuous variables with evidence of skewing). Within group changes over time (pre- to postintervention) were evaluated using chi-square or McNemar test (categorical variables), paired t tests (continuous variables), or Wilcoxon signed rank test (ordinal or continuous variables with evidence of skewing). Between-group differences over time were assessed using Repeated Measures ANOVA (multivariate tests). Distributions of all dependent variables were examined to ensure the assumptions of normality and sphericity were met and variables transformed as necessary. Between-group differences over time in categorical variables were assessed using logistic regression. Effect sizes were calculated using Cohen's *d *[67], with between-group effect size adjusted for differences in baseline values.

## 3. Results

Twenty (27%) of the 75 women enrolled in the parent trial met the IRLSSG four essential diagnostic criteria for RLS, with symptoms at least once per month (*n* = 10 yoga group, 10 film group participants). Only 2 (10%) of the 20 reported having received an RLS diagnosis; neither were taking RLS medications. Of those with RLS, 17 (8 yoga, 9 control) experienced symptoms at least once/week. Baseline characteristics of the participants with versus without RLS are given in Table 1. Relative to those without RLS, women with RLS demonstrated significantly poorer sleep quality, greater prevalence of insomnia (85% versus 38% for those with versus without RLS, *P* = 0.0002), shorter sleep duration (6.2 ± 0.3 versus 7.1 ± 0.2 hours, *P* = 0.002), and higher diastolic and systolic blood pressure (*P* < 0.005) and were more likely to report a history of depression (*P* < 0.01). Participants with RLS were less likely to specify a history of hormone replacement therapy (*P* = 0.03) and indicated a significantly higher number of pregnancies than those without RLS (*X* = 2.9 ± 0.5 versus 1.8 ± 0.2, resp., *P* = 0.02). Those with RLS also tended to report higher trait anxiety, greater mood impairment, and lower vigor (*P* < 0.1). Women with RLS did not differ from those without RLS in baseline ferritin levels (81 ± 10.5 versus 84.7 ± 8.4 ng/mL, resp., *P* = 0.8) or in the prevalence of low ferritin levels (< 50 ng/mL, 30% versus 36%, resp., *P* = 0.6). 

Of those 20 participants screening positive for RLS, 5 (25%) were African American, 65% had completed at least 4 years of college, 65% were employed at least part time, 35% were married, and 50% were pre-diabetic (fasting glucose 100–125 mg/dL). Participant age averaged 58.8 ± 0.9 years. As indicated in Table 2, treatment and control groups did not differ in demographic characteristics, or in baseline lifestyle factors, anthropometric characteristics, medical history, health profiles, prevalence of insomnia, or reported frequency of RLS symptoms. Mood and sleep profiles were also similar between the groups (*P* > 0.3, data not shown). Eighteen of the 20 participants with RLS completed the final assessment (8 yoga, 10 controls). Those leaving the study (one non-Hispanic white, one African American) dropped out during the first 2-3 weeks due to health reasons unrelated to the yoga program. Adherence in both the yoga and the educational film group was very good overall. Class attendance in the two groups was similar (mean attendance = 13.1 ± 1.4 versus 13.6 ± 0.8 classes (of 16 total classes) for the yoga versus film group participants, respectively, *P* = 0.75). Yoga group participants completed homework practice a mean of 4.1 ± 0.2 days/week, with an average 28.0 ± 3.3 minutes/practice session. Participant feedback on open-ended exit questionnaires was also positive, with participants of both groups expressing enthusiasm for their respective programs [57]. Of the 8 yoga group participants completing the study, all cited high satisfaction with the yoga program and all reported multiple benefits on their yoga logs and/or exit questionnaires, including increased strength, flexibility and mobility (*N* = 6), reduced pain (*N* = 3), enhanced energy and well-being (*N* = 6), increased feelings of peace, tranquility, and relaxation (*N* = 8), and greater body awareness (*N* = 4). While three yoga group participants indicated some mild, temporary muscle soreness in the first few weeks, no participants reported significant pain, discomfort or other adverse events in association with the yoga program. 

As illustrated in Table 3, yoga group participants showed significant reduction over time in prevalence of insomnia (*P* = 0.01) and significant improvement in sleep quality, both overall (*P* = 0.001) and in the domains of sleep duration (*P* = 0.02), efficiency (*P* = 0.01), disturbance (*P* = 0.03), and daytime dysfunction (*P* = 0.002). Reported average sleep duration increased from 5.7 ± 0.5 to 7.3 ± 0.5 hours (*P* = 0.001). Yoga participants also demonstrated significant, or marginally significant improvements in all but one domain of mood (*P* ≤ 0.08), as well as significant reductions in state anxiety, perceived stress, and both systolic and diastolic blood pressure (*P* < 0.05).

Despite limited study power, the yoga group demonstrated significantly greater improvement than controls in several domains of sleep quality, greater reductions in prevalence of insomnia, and greater increases in average sleep duration. Relative to controls, yoga group participants also showed significantly greater reductions in perceived stress, mood disturbance, state anxiety, and both systolic and diastolic blood pressure (all *P*'s ≤0.05). Adjusted intergroup effect sizes for psychosocial variables were large, with those for summary scores ranging from 1.9 for state anxiety to 2.6 for sleep quality (Table 3); intergroup effect sizes for blood pressure were also substantial, calculated as 0.9 for diastolic blood pressure and 1.25 for systolic blood pressure. Intent to treat analysis, using the conservative last value carried forward method to address missing data, did not appreciably alter these findings. Neither treatment expectancy scores nor change in physical activity or social support differed significantly between the two groups; moreover, adjustment for these variables did not materially alter the results, suggesting that these factors did not explain the observed between-group differences.

## 4. Discussion 

RLS is a common sleep and sensorimotor disorder associated with significant reductions in quality of life that are largely attributable to the substantial impairment in sleep and mood commonly accompanying RLS [4]. In light of the potentially serious side effects of existing pharmacological treatments for RLS, investigation of promising lifestyle and behavioral interventions is clearly warranted. To our knowledge, this is the first study to examine the effects of yoga in persons with RLS, and among the few trials to examine the potential benefits of any nonpharmacologic intervention for those suffering from this disorder. Findings of this preliminary RCT suggest that yoga can significantly improve sleep, enhance mood, reduce stress and anxiety, and decrease blood pressure in postmenopausal women with RLS, and thus may offer a promising new treatment modality for this population. Moreover, the overall excellent compliance, high participant satisfaction, and lack of adverse events observed in this study suggest that a gentle yoga program is both feasible and acceptable to older women with RLS. 

Consistent with our findings, a nonrandomized controlled trial in 14 dialysis patients with RLS reported significant improvements in sleep and mood in those completing a 16-week aerobic exercise program, with adjusted intergroup effect sizes ranging from 0.75 to 1.00 [37]. Likewise, a pre-post study of 25 outpatients with psychosocial impairment due to RLS reported significant, although more modest improvements in sleep and mood following 8 weeks of CBT (effect sizes 0.1–0.8) [39], improvements that appeared attributable at least in part to mindfulness, breathing, and other stress-reduction exercises. Although no published studies have assessed the effects of yoga on blood pressure in RLS patients, a number of controlled studies have reported yoga interventions to reduce blood pressure in other both healthy and chronically ill populations [41, 49, 68–70]. 

While we did not assess the effects of yoga specifically on symptoms of RLS, change in sleep and mood are endpoints recommended for use in clinical trials by the IRLSSG [7] and have been used in trials regarding the efficacy of dopamine agonists and other pharmaceutical treatments for RLS [71–76]. Of particular note, the improvements in sleep quality and mood observed following our 8-week yoga program appear comparable to those reported in recent pharmaceutical trials that used similar measures in RLS patients [71, 72, 74, 76, 77], suggesting that yoga could possibly provide a viable alternative to pharmaceutical therapy for some patients. Given these promising preliminary findings, larger controlled trials are warranted to investigate the potential utility of yoga as an adjunct or primary treatment for RLS. 

Only 10% of study participants screening positive for RLS had received a physician diagnosis, comparable to the low diagnostic rates generally reported in other, larger studies [78–80]. Consistent with previous research [81–83], participants with RLS reported higher parity and demonstrated significantly greater sleep deficits and mood disturbance than those without RLS, with 85% indicating insomnia at baseline and 50% reporting a history of depression. In agreement with several, but not all recent studies [21], RLS was associated with significantly higher blood pressure in this study. Participants with RLS were also less likely to report use of hormone replacement therapy (HRT) than those without RLS. Evidence from recent experimental studies in postmenopausal women suggest HRT may decrease complaints of restless legs [84], improve subjective sleep quality [85, 86], suppress muscle sympathetic nerve activity [87], and reduce nocturnal arousals [84], findings consistent with a possible protective effect of HRT for RLS. However, the relation between HRT and RLS remains unclear, with 2 epidemiological studies indicating positive, although nonsignificant, associations between RLS and HRT [88, 89], and a French study of 440 postmenopausal women with RLS reporting no relation of HRT use to symptom severity [90]. 

**Figure 1:**
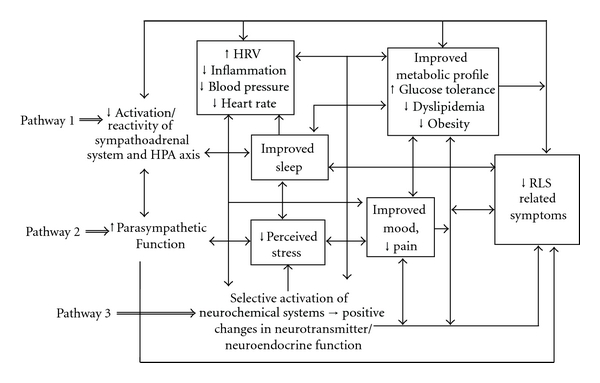
Possible pathways by which yoga may attenuate the distressing symptoms associated with RLS.

Although mechanisms underlying the improvements with yoga observed in this study remain speculative, yoga may benefit those with RLS via several possible interrelated pathways, illustrated in Figure 1. For example, by reducing the activation and reactivity of the sympathetic nervous system and the HPA axis, factors recently implicated in RLS etiology [9, 21, 25] and known to have strong, bidirectional relationships with sleep and mood [91, 92], yoga may attenuate RLS-associated pain and discomfort, reduce perceived stress and promote feelings of well-being, enhance sleep, lower muscle excitability, and foster positive downstream changes in metabolic status, neuroendocrine function, and inflammatory responses. Second, as illustrated in pathway 2, yoga may also alleviate the distressing symptoms of RLS by directly enhancing parasympathetic output, possibly via stimulation of the vagus nerve [93–95], and in this way, shift the autonomic nervous system balance from primarily sympathetic to parasympathetic [93, 95]. This, in turn, may enhance sleep and mood, reduce perceived pain, promote muscle relaxation, and lead to positive changes in cardiovagal function and related neuroendocrine, metabolic, and inflammatory responses. 

Third, findings of recent neuroimaging and neurophysiological studies [93, 96, 97] suggest that yogic practices may, by selectively activating specific neurochemical systems implicated in RLS, likewise promote beneficial changes in sympathetic/parasympathetic balance, in neuroendocrine function, in affect, sleep, and pain processing, and in related metabolic and inflammatory responses (pathway 3). For example, yogic practices have been shown to increase brain levels of dopamine [98], a neurotransmitter long implicated in the development of RLS and thought to play a key role in pain processing [99], sleep [100], motor control [101], and metabolic regulation [102, 103]. Yoga also increases GABA [97], an inhibitory neurotransmitter involved in the regulation of muscular excitability [104], mood [105], sleep [100], and pain processing [106]; the GABA agonist gabapentin, an anticonvulsant used in treating RLS [73, 77], as well as chronic pain, mood disorders, and insomnia [107–109], is thought to operate at least in part by increasing brain GABA [73, 77]. 

### 4.1. Strengths and Limitations

Strengths of this study include the community-based design, randomization of participants, between-group similarity in baseline characteristics, high participant retention and adherence, and a comparison condition designed to control for time, attention, and social interaction. Limitations of this pilot study are several. Sample sizes were small, reducing our power to detect between-group differences over time. However, despite limited power, we observed significant improvement in the yoga versus control group in several clinically important parameters, again arguing for a potentially powerful beneficial effect of the yoga program. Although the educational film program was designed to control for staff attention, setting, and class time, control group participants did not receive homework assignments. In addition, while assessors were blinded to participant treatment status, participants could not be masked. However, response to the educational film (control) program was enthusiastic, and both retention and compliance were excellent [57]. Treatment expectancies did not differ between-groups, and adjustment for treatment expectancies did not materially alter findings, suggesting that placebo effects did not explain the observed findings. Moreover, observed effect sizes were substantially larger than would be expected with placebo [110], and significantly greater than those observed in the controls. 

The study population was restricted to sedentary, overweight, postmenopausal women, and findings may therefore not be generalizable to other populations. RLS diagnosis was based on self-report, and some degree of diagnostic error is thus likely. However, affirmative response to all 4 essential criteria renders inclusion of mimics less likely [111]. Finally, the parent study was not designed specifically to address effects of yoga practice on RLS, and we did not measure change in RLS symptoms *per se.* However, as indicated above, we did screen for RLS at baseline and assess change in sleep quality and mood, which are outcomes that have been recommended for inclusion in clinical trials of RLS [7] and often used as secondary endpoints in pharmaceutical trials of RLS patients [112–116]. Significant sleep and mood impairment have been repeatedly documented in studies of RLS [7, 117, 118]. Sleep loss is the most common presenting complaint of patients seeking medical care for RLS [118, 119] and is thought to explain, in large part, the negative effects of RLS on health and quality of life [80]. 

## 5. Conclusions 

 These preliminary findings suggest that yoga may offer a safe, beneficial intervention for reducing sleep and mood disturbance, perceived stress, anxiety, and blood pressure in older women with RLS. Larger controlled trials are needed to confirm these benefits in this and other adult populations with RLS, examine the effects of yoga practice specifically on RLS symptoms and symptom severity, and to evaluate potential underlying mechanisms.

## Figures and Tables

**Table 1:** Baseline characteristics of participants with (*N* = 20) versus without (*N* = 55) symptoms of restless legs syndrome (RLS).

	Restless Legs Syndrome				
	No		Yes		
	Frequency/Mean	Percent/SE	Frequency/Mean	Percent/SE	*P*
Demographic and lifestyle characteristics					
Age in years (Mean, SE)	58.78	0.90	58.65	1.70	NS
Race/ethnicity					NS
Non-Hispanic White	45	81.82%	15	75.00%	
Minority	10	18.18%	5	25.00%	
Education					NS
<4 years college	13	23.64%	7	35.00%	
≥4 years college	42	76.36%	13	65.00%	
Employed	39	70.91%	13	65.00%	NS
Married/Cohabiting	27	49.09%	7	35.00%	NS
Mean daily intake of the following:					
Caffeine, mg (Mean, SE)	148.89	24.86	156.47	31.49	NS
Alcohol, g (Mean, SE)	8.45	2.93	20.13	11.14	NS
Health and reproductive history					
Reported history of:					
High blood pressure	14	25.45%	9	45.00%	0.10
Depression	10	18.18%	10	50.00%	0.008
Anxiety	8	14.55%	5	25.00%	NS
Hormone replacement therapy	11	20.00%	0	0.00%	0.03
Never pregnant	14	25.45%	2	10.00%	NS
Number of pregnancies	1.82	0.20	2.90	0.55	0.02
Sleep and psychosocial profiles					
* Sleep Quality (PSQI)*					
Sleep quality-Global	5.39	0.51	8.33	0.75	0.003
Sleep quality	0.61	0.15	1.30	0.29	0.03
Sleep latency	0.92	0.14	1.05	0.26	NS
Sleep duration	0.65	0.09	1.35	0.20	0.0005
Sleep efficiency	0.40	0.11	1.00	0.26	0.02
Sleep disturbance	1.24	0.07	1.67	0.11	0.003
Sleep medication use	0.22	0.06	0.55	0.21	0.04
Daytime dysfunction	1.29	0.10	1.65	0.15	0.06
Insomnia (PSQI > 5) (N, %)	21	38.18%	17	85.00%	0.0002
Average sleep duration in hours (Mean, SE)	7.10	0.15	6.18	0.25	0.002
* Anxiety (STAI) *					
Trait	34.65	1.29	38.95	1.94	0.08
State	30.35	1.18	32.74	2.45	NS
* Mood (POMS)*					
Total	8.96	4.26	23.89	9.47	0.09
Tension Anxiety	3.19	0.74	4.80	1.44	NS
Depression	7.09	1.10	11.50	2.82	0.08
Anger/hostility	5.27	0.70	8.26	2.47	NS
Vigor	16.87	0.93	13.55	1.30	0.06
Fatigue	8.28	0.90	10.35	1.45	NS
Confusion	1.76	0.60	2.80	1.17	NS
* Perceived stress (PSS)*	19.60	1.01	20.95	2.16	NS
* Social support (DSSI)*					
Social interaction	8.75	0.53	10.05	0.72	NS
Social support	18.34	0.38	17.79	0.75	NS
Blood Pressure					
Systolic (mmHg)	122.76	2.26	141.85	4.71	0.0001
Diastolic (mmHg)	71.35	1.22	78.75	2.57	0.005
Heart rate	64.82	1.28	66.65	2.62	NS
Anthropometrics					
Waist girth (cm)	103.83	2.20	108.46	2.97	NS
Hip girth (cm)	110.26	1.51	113.58	1.43	NS
Body mass index (kg/m^2^)	31.56	0.96	33.31	1.29	NS
Prediabetic (fasting glucose 100–125 mg/dL)	21	38.18%	10	50.00%	NS
Serum ferritin levels (ng/mL)(Mean, SE)	84.72	8.43	81.00	10.50	NS
Low ferritin level (<50 ng/mL)	20	36.36%	6	30.00%	NS

Abbreviations. DSSI: Duke Social Support Index.

NS: nonsignificant (*P* > 0.1).

POMS: Profile of Mood States.

PSQI: Pittsburgh Sleep Quality Index.

PSS: Perceived Stress Scale.

SE: standard error of the mean.

STAI: State-Trait Anxiety Inventory.

**Table 2:** Baseline characteristics of participants with restless legs syndrome (RLS) assigned to the yoga (*N* = 10) versus the educational film (control) group (*N* = 10).

	Treatment Group				
	Yoga		Educational Film (control)		
	Frequency/Mean	Percent/SE	Frequency/Mean	Percent/SE	*P*
Demographic and lifestyle characteristics					
Age in years (Mean, SE)	58.40	2.00	58.90	2.88	NS
Race/ethnicity					NS
Non-Hispanic White	8	80.00%	7	70.00%	
African American	2	20.00%	3	30.00%	
Education					NS
<4 years college	3	30.00%	4	40.00%	
≥4 years college	7	70.00%	6	60.00%	
Employed	7	70.00%	6	60.00%	NS
Married/Cohabiting	4	40.00%	3	30.00%	NS
Mean daily intake of the following:					
Caffeine (mg)	162.68	49.93	147.77	35.74	NS
Alcohol (g)	19.57	15.91	20.42	16.38	NS
Health history					
Reported history of:					
High blood pressure	4	40.00%	5	50.00%	NS
Depression	5	50.00%	5	50.00%	NS
Anxiety	2	20.00%	3	30.00%	NS
Prediabetic (fasting glucose 100–125 mg/dL)	5	50.00%	5	50.00%	NS
Serum ferritin levels (ng/mL)	81.40	15.35	80.56	15.32	NS
RLS symptoms					NS
1–3x/month	2	20.00%	1	10.00%	
At least 1x/week	8	80.00%	9	90.00%	
Obese (body mass index ≥30)	6	60.00%	5	50.00%	NS

Abbreviations. NS: nonsignificant (*P* > 0.5).

**Table 3:** Change over time in psychosocial and physiological profiles in sedentary, overweight postmenopausal women with RLS (*N* = 8 yoga, 10 controls).

	Yoga			Educational film (Control)			Between-group difference over time	
	Pre	Post	*P*	Pre	Post	*P*	*P*	Effect size¥
	(Mean ± SE)	(Mean ± SE)	(Mean ± SE)	(Mean ± SE)
Sleep quality, stress, and mood								
* Pittsburgh Sleep Quality Index (PSQI)*								
* Global*	8.71 ± 1.15	3.57 ± 0.53	0.001	9.25 ± 1.05	8.00 ± 0.93	NS	0.01	2.59
Sleep latency	0.88 ± 0.40	0.88 ± 0.35	NS	1.25 ± 0.41	1.50 ± 0.38	NS	NS	0.25
Sleep quality	1.38 ± 0.50	0.75 ± 0.41	NS	1.88 ± 0.40	1.38 ± 0.50	0.04	NS	0.11
Sleep duration	1.50 ± 0.38	0.50 ± 0.27	0.02	1.40 ± 0.40	1.30 ± 0.0.34	NS	0.04	1.18
Sleep efficiency	1.75 ± 0.41	0.63 ± 0.38	0.01	0.75 ± 0.37	0.38 ± 0.26	0.08	0.03	0.70
Sleep disturbance	1.71 ± 0.18	1.14 ± 0.14	0.03	1.75 ± 0.16	1.75 ± 0.25	NS	0.07	1.44
Sleep medication	0.13 ± 0.13	0.00 ± 0.00	NS	0.88 ± 0.35	0.63 ± 0.38	NS	NS	−0.33
Daytime dysfunction	2.00 ± 0.27	0.88 ± 0.13	0.002	1.50 ± 0.17	1.35 ± 0.21	NS	0.01	2.64
Average sleep duration in hours	5.72 ± 0.45	7.33 ± 0.53	0.001	6.40 ± 0.32	6.28 ± 0.30	NS	0.0001	−1.35
Prevalence of insomnia (PSQI > 5) (%)	87.50%	12.50%	0.01	100.00%	70.00%	NS	0.03	—
* Perceived Stress Scale*	24.00 ± 1.88	14.71 ± 2.75	0.006	18.44 ± 1.47	19.67 ± 1.39	NS	0.03	1.98
* Profile of Mood States*								
* Total*	24.00 ± 4.11	−12.50 ± 7.06	0.02	18.60 ± 2.71	24.11 ± 8.96	NS	0.02	2.35
Tension/Anxiety	5.75 ± 1.28	0.88 ± 1.69	0.07	4.22 ± 0.53	5.78 ± 1.55	NS	0.05	1.78
Confusion	3.43 ± 2.28	0.00 ± 0.49	NS	1.50 ± 1.45	2.00 ± 1.67	NS	0.09	2.84
Depression	13.25 ± 0.73	2.88 ± 1.23	0.001	11.22 ± 1.03	11.11 ± 1.87	NS	0.06	4.97
Anger/Hostility	5.14 ± 0.64	2.29 ± 0.67	0.02	7.33 ± 0.78	9.78 ± 1.30	0.06	0.06	2.93
Vigor	12.50 ± 1.08	19.88 ± 2.41	0.015	16.33 ± 0.91	15.67 ± 0.98	NS	0.02	−2.63
Fatigue	13.29 ± 1.95	7.43 ± 2.95	0.08	8.22 ± 1.20	9.33 ± 1.51	NS	0.05	1.26
* State-Trait Anxiety Inventory *								
State	33.29 ± 2.59	26.14 ± 2.22	0.05	28.13 ± 2.44	32.75 ± 3.37	0.05	0.002	1.87
Trait	39.71 ± 3.90	33.43 ± 4.02	NS	36.38 ± 2.77	34.88 ± 3.53	NS	NS	0.43
* Duke Social Support Index*								
Social Interaction	10.00 ± 1.33	10.00 ± 1.02	NS	10.75 ± 1.13	11.00 ± 0.89	NS	NS	NA
Social Support	17.17 ± 1.19	19.50 ± 0.43	NS	18.38 ± 0.71	18.13 ± 0.69	NS	NS	NA
Heart rate and blood pressure								
Average heart rate (supine)	68.13 ± 3.48	65.00 ± 3.64	NS	66.80 ± 4.56	64.60 ± 3.42	NS	NS	0.09
Systolic blood pressure	145.75 ± 9.80	125.50 ± 3.24	0.04	139.93 ± 6.44	131.13 ± 4.51	NS	0.05	1.25
Diastolic blood pressure	83.13 ± 4.36	73.75 ± 3.68	0.02	74.40 ± 3.42	73.80 ± 2.59	NS	0.03	0.89
Anthropometrics								
Waist (cm)	108.95 ± 5.79	106.91 ± 4.78	NS	108.60 ± 4.63	110.68 ± 4.74	NS	NS	0.25
Weight (kg)	88.83 ± 4.33	87.67 ± 4.30	NS	83.56 ± 3.66	84.26 ± 4.12	NS	NS	0.15
Body mass index (kg/m^2^)	33.59 ± 2.13	32.96 ± 1.74	NS	32.27 ± 1.95	32.30 ± 2.15	NS	NS	0.11

¥Between-group effect size (Cohen's *d*), adjusted for differences in baseline values, NS: Non-significant (*P* > 0.10).
